# The crosstalk between cholangiocytes and hepatic stellate cells promotes the progression of epithelial-mesenchymal transition and periductal fibrosis during *Clonorchis sinensis* infection

**DOI:** 10.1186/s13071-024-06236-2

**Published:** 2024-03-22

**Authors:** Junyeong Yi, Ji Hoon Jeong, Jihee Won, Seok Chung, Jhang Ho Pak

**Affiliations:** 1 Department of Biochemistry, Asan Medical Institute of Convergence Science and Technology (AMIST), University of Ulsan College of Medicine and Asan Medical Center (AMC), 88 Olympic-Ro 43-Gil, Songpa-gu, Seoul, 05505 Republic of Korea; 2 School of Mechanical Engineering, Korea University, 145 Anam-Ro, Seongbuk-gu, Seoul, 02841 Republic of Korea

**Keywords:** *Clonorchis sinensis*, Excretory-secretory products, Cholangiocytes, Hepatic stellate cells, Intercellular crosstalk, Epithelial-mesenchymal transition, Periductal fibrosis

## Abstract

**Background:**

*Clonorchis sinensis* infection is one of the risk factors that provokes chronic inflammation, epithelial hyperplasia, periductal fibrosis and even cholangiocarcinoma (CCA). Disrupted or aberrant intercellular communication among liver-constituting cells leads to pathological states that cause various hepatic diseases. This study was designed to investigate the pathological changes caused by *C. sinensis* excretory-secretory products (ESPs) in non-cancerous human cell lines (cholangiocytes [H69 cell line] and human hepatic stellate cells [LX2 cell line]) and their intercellular crosstalk, as well the pathological changes in infected mouse liver tissues.

**Methods:**

The cells were treated with ESPs, following which transforming growth factor beta 1 (TGF-β1) and interleukin-6 (IL-6) secretion levels and epithelial-mesenchymal transition (EMT)- and fibrosis-related protein expression were measured. The ESP-mediated cellular motility (migration/invasion) between two cells was assessed using the Transwell and three-dimensional microfluidic assay models. The livers of *C. sinensis*-infected mice were stained using EMT and fibrotic marker proteins.

**Results:**

Treatment of cells with ESPs increased TGF-β1 and IL-6 secretion and the expression of EMT- and fibrosis-related proteins. The ESP-mediated mutual cell interaction further affected the cytokine secretion and protein expression levels and promoted cellular motility. N-cadherin overexpression and collagen fiber deposition were observed in the livers of *C. sinensis*-infected mice.

**Conclusions:**

These findings suggest that EMT and biliary fibrosis occur through intercellular communication between cholangiocytes and hepatic stellate cells during *C. sinensis* infection, promoting malignant transformation and advanced hepatobiliary abnormalities.

**Graphical Abstract:**

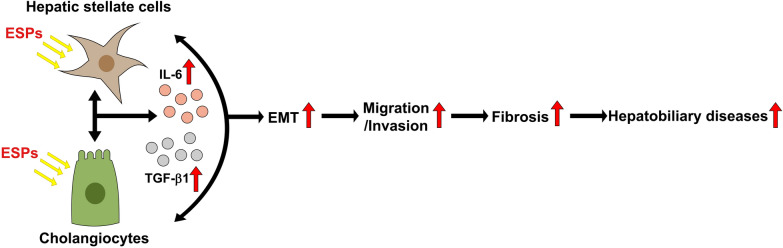

## Background

The liver comprises various cell types, including hepatocytes, intrahepatic cholangiocytes, Kupffer cells, liver sinusoidal endothelial cells (LSECs), oval cells and hepatic stellate cells (HSCs). These organizing cells are responsible for proper liver functions, including nutrient metabolism and detoxification, synthesis and transport of bile juice, an immune response against pathologic conditions, regulation of hepatic blood flow and fibrosis in response to liver damage. They regulate and communicate with each other to maintain the liver’s integrity and homeostasis [1, 2]. Disruption of intercellular crosstalk induces a pathological state, resulting in cell overactivation. For example, cholangiocytes are quiescent in the normal physiological condition; however, they are activated during bile duct disorders and secrete various cytokines, chemokines and hormones. These numerous mediators from reactive cholangiocytes affect other neighboring cells in a paracrine manner and trigger the activation of pathological signaling processes by altering the gene expression of surrounding cells. In response to liver damage, activated HSCs increase the synthesis of extracellular matrix (ECM) proteins (collagen type I (COL1), α-smooth muscle actin [α-SMA] and fibronectin), which play pivotal roles in hepatic fibrosis [3].

Cholangiocarcinoma (CCA) is characterized by aggressive malignancy of the biliary epithelia with enhanced invasiveness and metastasis. Established risk factors for CCA include exposure to toxins, hepatolithiasis and parasitic infections [4]. Infection with liver flukes, such as *Opisthorchis viverrini* and *Clonorchis sinensis,* is one of the major risk factors for CCA in East and Southeast Asian countries. the International Agency for Research on Cancer has listed these liver flukes as definite Group 1 biological carcinogenic agents [5]. Carcinogenesis associated with chronic liver fluke infection results from mechanical injury of biliary epithelia caused by the feeding and migratory activities of the worms, immunopathology due to infection-related inflammation and the direct effect of their excretory-secretory products (ESPs; a complex mixture of proteins and other metabolites), which are continuously released from the tegument and excretory openings of the flukes [6]. These combined physical and chemical irritations provoke inflammation, adenomatous hyperplasia, granuloma formation and periductal fibrosis with subsequent malignant transformation, creating vulnerability to a tumorigenic environment [7]. In an animal model, mass-forming CCA developed in Syrian golden hamsters that received *O*. *viverrini* or *C*. *sinensis* metacercariae together with a subcarcinogenic dose of *N*-nitrosodimethylamine, and the CCA tissues could be categorized into time-dependent well, moderately and poorly differentiated types [8, 9].

Treatment of cells with liver fluke ESPs has been used to explore in vitro infection-associated pathological processes and dysregulation. For example, in one study, the exposure of human CCA cells (HuCCT1) to *C. sinensis* ESPs triggered the enzymatic generation of free radicals, subsequently causing nuclear factor kappa B (NF-κB)-mediated inflammation [10]. ESPs of *O*. *viverrini* are internalized in cholangiocytes (H69 cells) through endocytosis pathways, stimulating cell proliferation and proinflammatory cytokine production [11]. Differential transcriptional and proteomic profiles of *C. sinensis* ESP-treated HuCCT1 cells showed that the identified genes/proteins were involved in cell structure/architecture, metabolism, redox homeostasis, proteolysis and signal transduction, demonstrating the multiple pathophysiological effects of ESPs on host cells [12, 13]. Another study showed that microRNAs involved in proliferation and inhibition of tumor suppression were dysregulated in HuCCT1 and H69 cells exposed to ESPs, indicating that ESP-triggered pathological signal cascades commonly occur in cancerous and non-cancerous bile duct epithelial cells [14].

Interactions between heterogenous liver-constituting cells have been recognized to affect liver function, homeostasis and disease progression. Co-culture of hepatocytes and HSCs was found to promote hepatocyte migration and changes in proinflammatory and angiogenic microenvironments [15]. Capillary-like sprouts were formed in co-cultured spheroids of LSECs and activated HSCs, indicating paracrine interactions between HSCs and endothelial cells [16]. Transwell co-cultures of an immature cholangiocyte line with myofibroblastic cells enhanced cell proliferation and viability in both cell types, which was mediated by each other’s Hedgehog signaling pathway [17]. With regard to liver fluke infection, *C. sinensis* ESP application promoted the invasion of HuCCT1 cells into the biliary ductal plate formed by H69 cells, as assessed using a three-dimensional (3D) microfluidic co-culture assay [18]. In addition, activated HSCs induced proliferation and migration/invasion of hepatocellular carcinoma (HCC) cells, suggesting that the HSC-HCC cell interaction may contribute to HCC progression and tumor differentiation [19].

In the present study, *C. sinensis* ESP-associated changes in inflammatory cytokines and protein expressions related to epithelial-mesenchymal transition (EMT) and fibrosis in non-cancerous liver-constituting cell lines (cholangiocytes [H69 cell line] and HSCs [LX2 cell line]) were examined. The mutual effects of these cells on the EMT, fibrosis and motility mediated by ESP treatment were also studied. In addition, a monolayer and a 3D co-culture system were used to investigate the pathological interactions between these two cells. In animal models, N-cadherin (N-cad) expression and collagen fiber deposition were examined in *C. sinensis*-infected mouse livers.

## Methods

### Materials

Cell culture medium components were purchased from Thermo Fisher Scientific (Waltham, MA, USA) unless otherwise indicated. The polyclonal antibodies used in the study were: (i) STAT3 (cat. no. 4904), phospho-STAT3 (pSTAT; cat. no. 3l9145), Smad2/3 (cat. no. 3102), phospho-Smad2/3 (pSmad2/3; cat. no. 8828), Smad4 (cat. no. 38454) and Snail (cat. no. 3879) from Cell Signaling Technology, Inc. (Danvers, MA, USA); (ii) E-cadherin (E-cad; cat. no. 610182) from BD Biosciences (San Jose, CA, USA); (iii) N-cad (cat. no. sc-59987) from Santa Cruz Biotechnology (Santa Cruz, CA, USA); (iv) α-SMA (cat. no. ab5694) and fibronectin (cat. no. ab2413) from Abcam (Cambridge, UK); (v) glyceraldehyde-3-phosphate dehydrogenase (GAPDH; cat. no. A300-641A) and horseradish peroxidase (HRP)-conjugated secondary antibodies from Bethyl Laboratories, Inc. (Montgomery, TX, USA). Other chemicals were purchased from Sigma-Aldrich (St. Louis, MO, USA).

### Cell culture and ESP treatment

H69 cells were cultured in Dulbecco’s modified Eagle’s medium (DMEM) and DMEM/F12 containing 10% fetal bovine serum (FBS), 100 μg/ml streptomycin, 100 U/ml penicillin, 5 μg/ml insulin, 5.5 μM epinephrine, 5 μg/ml transferrin, 2.0× 10^−9^ M triiodothyronine, 1.8 × 10^−4^ M adenine, 1.1 μM hydrocortisone and 1.6 μM epidermal growth factor (EGF). HSCs of cell line LX2 were cultured in DMEM containing 10% FBS, 100 μg/ml streptomycin and 100 U/ml penicillin. The cells of both lines were incubated at 37 °C in a humidified 5% CO_2_ incubator. For ESP treatments, cells were seeded on 60-mm culture dishes and incubated for 24 h under standard conditions. The cells were incubated in a serum-free medium for 3 h followed by exposure to 1.6 μg/ml ESPs for 24 h.

### Preparation of the LX2 conditional medium

To produce the conditional medium, LX2 cells were seeded on 100-mm culture dishes and incubated for 24 h under standard conditions. The next day, the medium was replaced with serum-free medium containing 1.6 μg/ml ESPs and the cells incubated for a further 24 h. The medium was collected and centrifuged at 3000 rpm for 5 min to remove cell debris. The medium in which LX2 cells had been grown in the presence of ESPs, referred to as the LX2-conditional medium with ESPs (LX2-ESPs-CM), was aliquoted and stored at − 80 °C until use.

### Enzyme-linked immunosorbent assay

The secretion levels of transforming growth factor beta 1 (TGF-β1) and interleukin-6 (IL-6) were quantified using a commercial human enzyme-linked immunosorbent assay (ELISA) kit (Enzo Life Science, Inc., Farmingdale, NY, USA) according to the manufacturer’s protocol. The culture supernatants were added to each well and incubated with the reaction buffer, following which absorbance was measured at 450 nm using an ELISA reader (Spectra MAX340PC; Molecular Devices LLC, San Jose, CA, USA). The level of each cytokine was calculated using human recombinant TGF-β1 and IL-6 standard curves.

### Immunoblot analysis

Cells were washed three time with phosphate-buffered saline (PBS) and lysed with RIPA buffer (Sigma-Aldrich) containing phosphatase and protease inhibitor cocktails. Protein concentrations were determined using the BCA Protein Assay kit (Thermo Fisher Scientific). Total soluble proteins were separated by sodium dodecyl sulfate-polyacrylamide gel electrophoresis (SDS-PAGE) and electrophoretically transferred to nitrocellulose membranes (GE Healthcare Biosciences, Uppsala, Sweden). The membranes were blocked using 5% skim milk, followed by incubation with primary antibodies overnight at 4 °C. After incubation with appropriate HRP-conjugated secondary antibodies, the membranes were treated with enhanced chemiluminescence (ECL; Dongin LS, Seoul, Korea). Immunoreactive bands were detected using an Image-Quant LAS 500 biomolecular imager (GE Healthcare Biosciences).

### Transwell co-culture assay

Cell migration and invasion assays were performed in a 24-well microplate with 8-μm pore size cell culture inserts (BD Falcon tubes [catalog no. 353097]; BD Biosciences, Franklin Lakes, NJ, USA). For the migration assay, H69 and LX2 cells were seeded in the upper inserts and lower wells, or vice versa, on the microplate and co-incubated for 24 h. The medium in the lower well was substituted with a medium containing 1.6 μg/ml ESPs and further co-incubated for 1 and 3 days. The upper inserts were removed, washed twice with PBS and fixed with 70% methanol for 10 min. After washing twice with distilled water, the upper inserts were stained with 0.2% crystal violet for 10 min. The non-migrated cells on the upper surface of the inserts were carefully removed using a cotton swab. The stained cells were captured using an upright microscope (Eclipse Ci; Nikon, Tokyo, Japan), and the number of cells was counted using three randomly selected fields per insert. For the invasion assay, the upper inserts were pre-coated with Matrigel (BD Biosciences) and followed by the same procedures for the migration assay.

### Immunofluorescence staining

The cells were washed three times with PBS and fixed with 4% paraformaldehyde for 15 min, After further washing with PBS, the cells were permeabilized with 0.1% Triton X-100 for 3 min and blocked with 1% bovine serum albumin for 1 h. Then, the cells were incubated with primary antibodies against N-cad (1:200 dilution) and fibronectin (1:200 dilution) overnight at 4 °C. After washing with PBS three times, the cells were incubated with anti-mouse or anti-rabbit secondary antibodies conjugated with Alexa Fluor 488 (1:1000 dilution; Invitrogen, Thermo Fisher Scientific) for 1 h. The cells were counterstained with 4′,6-diamidino-2-phenylindole (DAPI, 1:1000 dilution; Sigma-Aldrich) for 5 min and mounted in fluorescence mounting solution. Cells cultured in the 3D microfluidic devices were also stained with fibronectin and DAPI as reported previously [20]. Cell images were obtained using a fluorescence microscope (Axio Observer Z1; Carl Zeiss, Oberkochen, Germany). Fluorescence intensity was calculated using ImageJ software. The total cell fluorescence (CTCF) values were corrected using the following calculation: CTCF = integrated density − (area of selected cells × mean fluorescence of background).

### Preparation of the 3D microfluidic devices

The microfluidic devices were prepared as described previously [18] using a soft lithography procedure. Polydimethylsiloxane (PDMS) was poured into an SU-8-patterned wafer template. Once solidified, the devices were punched to make ports for injecting the hydrogel and cell suspension. The devices were then sterilized and bonded to a glass coverslip using oxygen plasma. Microchannels in the devices were filled with poly-D-lysine and washed with deionized distilled water. The devices were then dried in an oven and kept sterile until use.

### 3D culture of H69 and LX2 cells using a microfluidic device and ESP treatment

Unpolymerized COL1 hydrogel solution (2.0 mg/ml, pH 7.4) was injected into the two central ports of the microfluidic device and the device then incubated at 37 °C for 30 min in a humidified chamber for hydrogel polymerization. LX2 cells were grown in H69 media containing 10% FBS and EGF for 2 days to stabilize the cells under culture conditions until cell seeding. LX2 cells (1 × 10^6^) were suspended in H69 media and loaded into one medium port. After filling, the devices were vertically positioned for 2 h in a 5% CO_2_ incubator at 37 °C to induce cell attachment to the hydrogel. The next day, the medium inside the channel was substituted with a conditional medium (FBS-free, EGF-depleted). The opposite channel was filled with H69 cells (5 × 10^5^) suspended in H69 media and placed vertically in an incubator for 2 h. The channel medium in which the H69 cells were suspended was also substituted with a conditional medium. ESPs diluted in a conditional medium at a final concentration of 4 μg/ml were treated to the H69 or LX2 channel.

### Preparation of *C. sinensis* metacercariae and infection

Metacercariae of *C. sinensis* were obtained from naturally infected freshwater fish (*Pseudorashora para*) in an endemic area in Korea. Fish were ground and digested in artificial gastric juice (0.4% pepsin, pH 1.0; MP Biochemicals Co., Solon, OH, USA). The coarse matter remaining after digestion was filtered using a 1-mm-diameter mesh sieve. The filtrate was washed several times with 0.85% saline. Metacercariae were obtained under a dissecting microscope and placed in PBS containing antibiotics and antimycotics at 4 °C until use. Male FVB/NJ mice were orally infected with approximately 30 *C. sinensis* metacercariae. The mice were fed an appropriate amount of sterilized commercial diet and water ad libitum without further treatment. The mice were euthanized sequentially after 1 and 3 months.

### Immunohistochemistry

Paraffin-embedded liver tissue sections were prepared as described previously [21]. Tissue sections (thickness: 5 μm) were deparaffinized with xylene, rehydrated through a graded ethanol series and rinsed with distilled water. After antigen retrieval and inactivation of endogenous peroxidase activity, the sections were incubated with the primary antibody against N-cad (1:200 dilution) overnight at 4 °C, then incubated with a secondary antibody and treated using the ABC-HRP kit (VECTASTAIN®, Vector Laboratories, Newark, CA, USA). Immunostaining of N-cad was visualized using the DAB Peroxidase Substrate Kit (Vector Laboratories). Sections were briefly counterstained with Harris hematoxylin (Sigma-Aldrich). For visualization of collagenous connective tissue fibers, the slides were stained using a trichrome stain kit (catalog no. ab150686; Abcam) according to the manufacturer’s protocol. Briefly, sections were deparaffinized, preheated with Bouin’s Fluid and incubated with Weigert’s Iron hematoxylin and Biebrich Scarlet/Acid Fuchsin solutions. The sections were then differentiated in a phosphomolybdic/phosphotungstic acid solution and incubated with Aniline Blue and acetic acid solutions. The stained slides were dehydrated, cleared and sealed with a mounting medium. Images were taken using an upright microscope (model Eclipse Ci; Nikon).

### Statistical analysis

All data were presented as the mean ± standard error (SE) of three independent experiments. Statistical analyses were performed using SigmaPlot software (version 12.0; Jandel Scientific, San Rafael, CA, USA) and GraphPad Prism software (GraphPad Software, Inc., San Diego, CA, USA). The Student’s t-test or one-way analysis of variance (ANOVA) was used to assess differences between groups. Statistical significance was set at *P* < 0.05.

## Results

### Effect of ESPs on cytokine levels, cell morphology and cytokine-, EMT- and fibrosis-related protein expression in H69 and LX2 cells

**Fig. 1:**
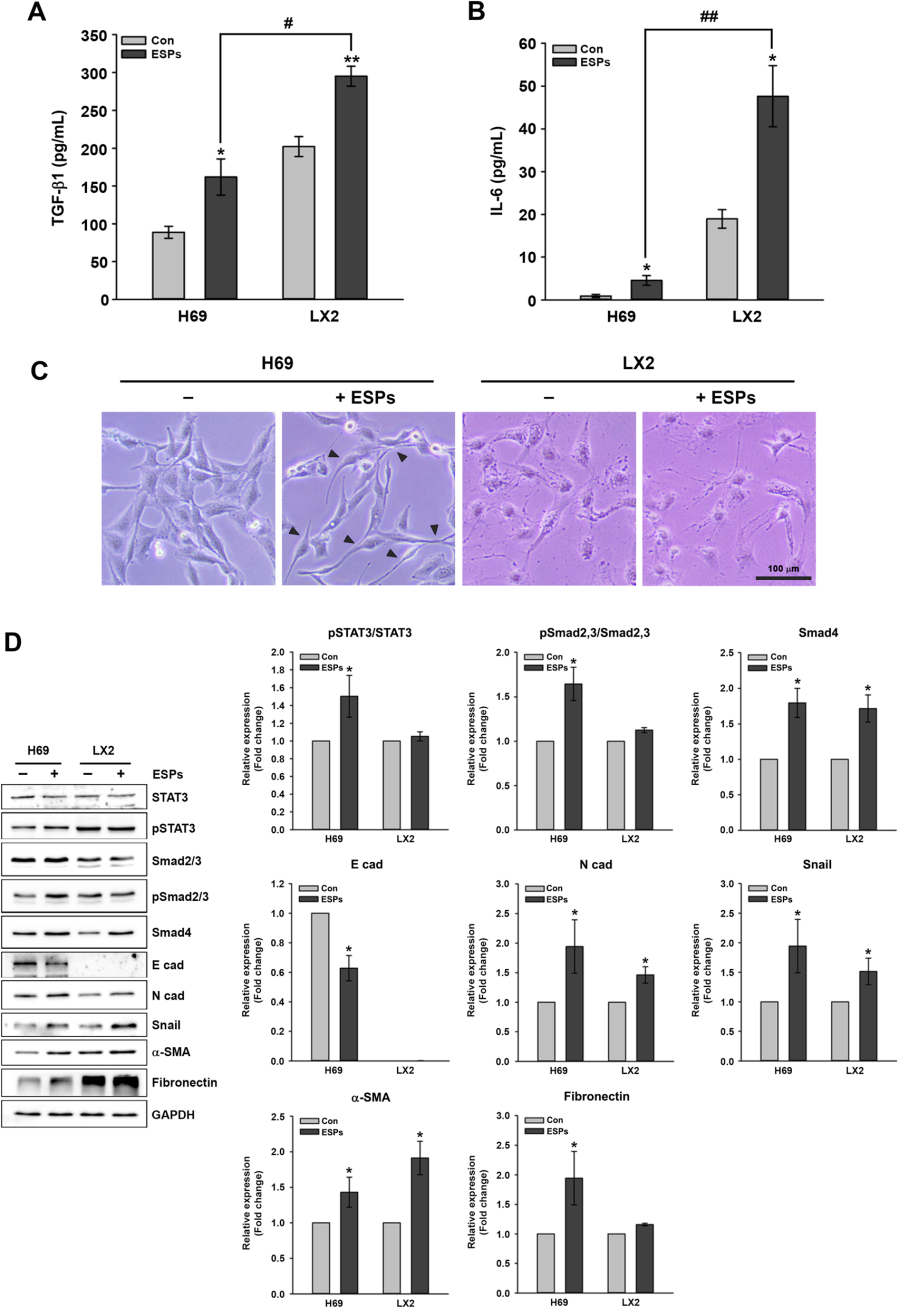
Effects of ESPs on the secretion of inflammatory cytokines (TGF-β1 and IL-6), cell morphology and EMT- and fibrosis-related protein expression. H69 and LX2 cells were treated with 1.6 μg/ml ESPs for 24 h. The supernatants were harvested, and the secretion levels of the cytokines TGF-β1 (**A**) and IL-6 (**B**) were measured using an enzyme-linked immunosorbent assay. Data represent the means ± SE for 3e independent experiments. Single asterisk (*)/single hash sign (#) indicate significant difference at *P* < 0.05; double asterisks (**)/double hash signs (##) indicate significant difference at *P* < 0.01, with the asterisks referring to control vs ESPs-treated cells, and the hash signs referring to ESP-treated H69 vs ESP-treated LX2 cells. **C** Representative light microscope images of ESP-treated cells. Black arrowheads indicate the morphology of fibroblast-like cells. Scale bar: 100 μm, original magnification: ×40. **D** Representative immunoblotting showing the expression of TGF-β1-, IL-6-, EMT- and fibrosis-related proteins. Protein bands were quantified using densitometry, and GAPDH was used as a normalization loading control. Data in graphs are represented as fold changes relative to the untreated control and expressed as the mean ± SE of 3 independent experiments. Single asterisk indicates a significant difference at *P* < 0.05 for untreated control vs ESP-treated cells. Con, Untreated control; EMT, epithelial-mesenchymal transition; ESP, excretory-secretory products; GAPDH, glyceraldehyde-3-phosphate dehydrogenase; IL, interleukin; SE, standard error; TGF-β1, transforming growth factor beta 1

Inflammatory cytokine secretion levels have been reported to be significantly increased in ESP-treated HuCCT1 cells [22]. To examine the effects of ESPs on the secretion of inflammatory cytokines in non-cancerous cells, we measured the TGF-β1 and IL-6 levels in ESP-treated H69 and LX2 cells and found that the secretion levels of both cytokines increased significantly in the ESP-treated H69 and LX2 cells compared with the untreated control (TGF-β1: t-test, *t*_(4)_ = − 2.889, *P* = 0.0446 and *t*_(4)_ = − 5.013, *P* = 0.00742, respectively; IL-6: t-test, *t*_(4)_ = − 3.063, *P* = 0.0375 and *t*_(4)_ = − 3.836, *P* = 0.0185, respectively). The basal levels of both cytokines in LX2 cells were higher than those in H69 cells. The ESPs induced increases in the TGF-β1 and IL-6 secretion levels of LX2 cells by approximately (~) 1.9- and  ~ tenfold, respectively, compared with those of H69 cells (TGF-β1: t-test, *t*_(4)_ = − 4.883, *P* = 0.00814; IL-6: t-test, *t*_(4)_ = − 5.944, *P* = 0.00402) (Fig. 1A, B).

We then investigated the morphological changes in the cell lines when exposed to ESPs. ESP-treated H69 cells had a fibroblast-like morphology compared with the untreated control. In contrast, no obvious morphological changes were observed in the ESP-treated LX2 cells (Fig. 1C). Assessment of the effects of ESPs on the expression of TGF-β1-, IL-6-, EMT- and fibrosis-related proteins using immunoblot analyses revealed that the related proteins were STAT3 and pSTAT3 for IL-6; Smad2/3, pSmad2/3 and Smad4 for TGF-β1; E-cad, N-cad and Snail for EMT; and α-SMA and fibronectin for fibrosis. As shown in Fig. 1D, the levels of pSTAT3 (active form of STAT3), pSmad2/3 (active form of Smad2/3) and Smad4 expression in ESP-treated H69 cells were increased by  ~ 1.5-,  1.6- and  ~ 1.8-fold, respectively, relative to that of untreated control (pSTAT3: t-test, *t*_(4)_ = − 2.801, *P* = 0.0488; pSmad2/3: t-test, *t*_(4)_ = − 3.457, *P* = 0.0259; Smad4: t-test, *t*_(4)_ = − 3.893, *P* = 0.0176). No significant changes in pSTAT3 and pSmad2/3 expression were observed in ESP-treated LX2 cells, but Smad4 expression was elevated by  ~ 1.7-fold (Smad4: t-test, *t*_(4)_ = − 3.746, *P* = 0.02). Additionally, E-cad expression levels were markedly reduced by  ~ 0.6-fold in the ESP-treated H69 cells compared with the untreated control, whereas the expression levels of N-cad, Snail, α-SMA and fibronectin were increased by  ~ 1.9-,  ~ 1.8-,  ~ 1.4- and  ~ 1.8-fold, respectively (E-cad: t-test, *t*_(4)_ = 4.316, *P* = 0.0125; N-cad: t-test, *t*_(4)_ = − 2.996, *P* = 0.0401; Snail: t-test, *t*_(4)_ = − 2.863, *P* = 0.0458; α-SMA: t-test, *t*_(4)_ = − 2.782, *P* = 0.0497; fibronectin: t-test, *t*_(4)_ = − 3.146, *P* = 0.0346). E-cad expression was undetectable in untreated and ESP-treated LX2 cells. Although the fibronectin expression level was unchanged, the expression levels of other proteins, such as N-cad, Snail and α-SMA were increased by ~ 1.4-, ~ 1.5- and ~ 1.9-fold, respectively, in response to ESPs (N-cad: t-test, *t*_(4)_ = − 3.328, *P* = 0.0292; Snail: t-test, *t*_(4)_ = − 2.956, *P* = 0.0417; α-SMA: t-test, *t*_(4)_ = − 3.872, *P* = 0.018). The basal level of fibronectin expression in LX2 cells appeared to be higher than that in H69 cells. Altogether, these results indicated that ESPs elevated TGF-β1 and IL-6 secretion levels and promoted EMT and fibrosis progression in H69 and LX2 cells.

### Effect of ESPs on migration and invasion in co-cultured H69 and LX2 cells

**Fig. 2:**
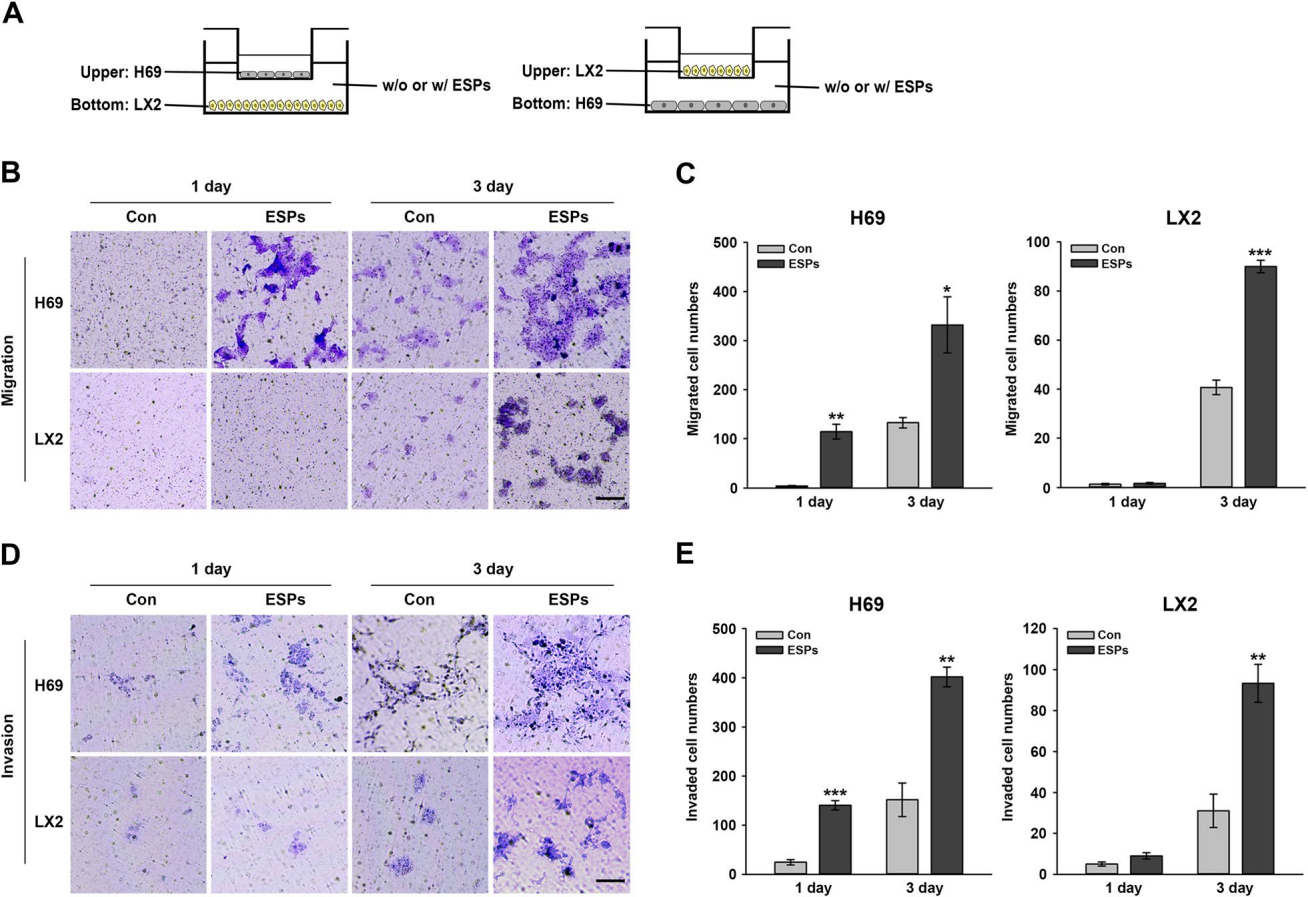
ESP-induced migration (non-coated) and invasion (Matrigel-coated) of H69 and LX2 cells co-cultured in a Transwell cell culture insert. **A** Schematic of co-cultured H69 and LX2 cells in a Transwell insert. Cells seeded on a Transwell cell culture insert were cultured for 24 h, following which medium supplemented with 1.6 μg/ml ESPs was added to the lower chamber and cells further incubated for 1 and 3 days. **B**,** D** Cell migration (**B**) and invasion (**D**) abilities were quantified by counting crystal violet-stained cells outside the porous membranes. Scale bar: 100 μm; original magnification: ×200. **C**,** E** Data in ** B** and** D** are represented in** C** and** E**, respectively, as the number of stained cells in three random fields using ImageJ software. Values are presented as the mean ± SE. Asterisks indicate significant a difference at *P < 0.05, **P < 0.01 and ***P < 0.001, compared with the untreated control. Con, Untreated control; ESPs, excretory-secretory products; SE, standard error

It has been reported that ESPs provoke cellular migratory and invasive potential in different CCA cell lines [23]. This result prompted our investigation on whether ESPs promoted crosstalk-mediated motility between non-cancerous cells under a co-cultured condition. H69 and LX2 cells were seeded in a Transwell chamber (H69 cells on an upper chamber/LX2 cells on a lower chamber, or vice versa) for 24 h, following which the lower chamber medium was substituted with a medium containing ESPs and the cells incubated for a further for 1 and 3 days (Fig. 2A). The migrating or invasive cells were stained with crystal violet and observed under a light microscope (Fig. 2B, D). One day after ESP treatment, the number of migrating H69 cells was ~ 20-fold higher than that of the untreated control, whereas no LX2 cell migrated (t-test, *t*_(4)_ = − 7.312, *P* = 0.00186). Three days after ESP treatment, the number of migrating H69 and LX2 cells increased by ~ 2.5- and ~ 2.3-fold, respectively, relative to that of the untreated control (t-test, *t*_(4)_ = − 3.438, *P* = 0.0264 and *t*_(4)_ = − 12.691, *P* < 0.001, respectively) (Fig. 2C). The number of invasive H69 cells was ~ 7 fold higher than that of the untreated control 1 day after ESP treatment, but no invasive LX2 cells were detectable (t-test, *t*_(4)_ = − 10.684, *P* < 0.001). At 3 days after treatment, the number of invasive cells was increased by ~ 2.3- and ~ 2.5-fold in H69 and LX2 cells, respectively, compared with that of the untreated control (t-test, *t*_(4)_ = − 6.3, *P* = 0.00325 and *t*_(4)_ = − 5.054, *P* = 0.00721, respectively) (Fig. 2E). Taken together, these results indicated that ESP-induced LX2 cells affected the migration and invasion of H69 cells relatively early. These ESP-induced abilities were eventually enhanced in both cells at a later time, suggesting involvement in the communication between H69 and LX2 cells.

### Elevation of cytokine secretion levels and progression of EMT and fibrosis in H69 cells cultured in LX2-ESPs-CM

**Fig. 3:**
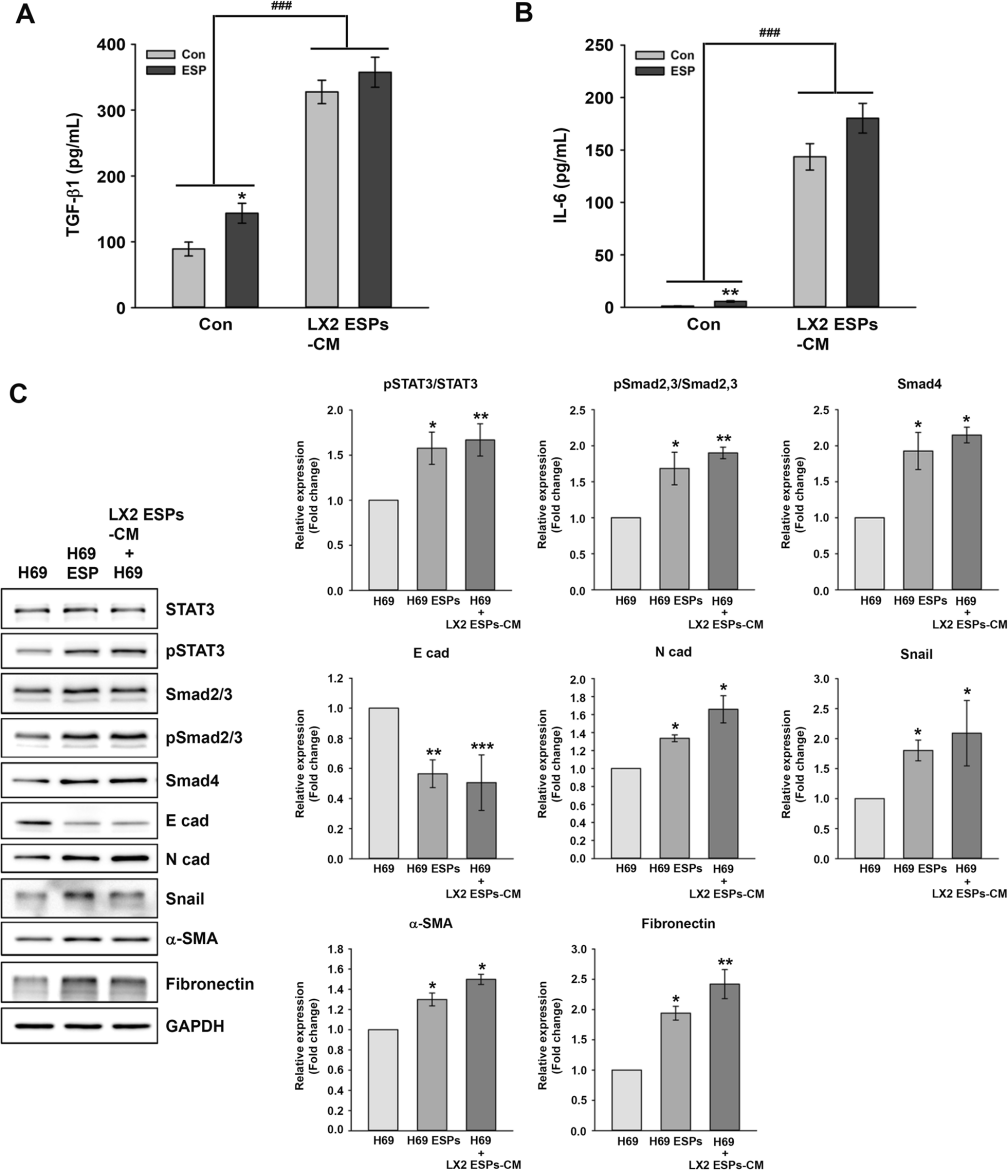
Effects of LX2-ESPs-CM on the secretion of inflammatory cytokines and the expression of EMT-, and fibrosis-related proteins in H69 cells. H69 cells were incubated for 24 h in H69 media as a control or in the media in which LX2 cells were grown in the presence of ESPs (LX2-ESPs-CM) for 24 h, as described in the Methods section. **A**, **B** The effect of LX2-ESPs-CM on TGF-β1 and IL-6 secretion levels of H69 cells, measured using an enzyme-linked immunosorbent assay. Data are presented as the means ± SE of 3 independent experiments. Asterisks indicate a significant difference at *P < 0.05 and **P < 0.01, compared with the untreated control; the triple hash signs indicate a significant difference at ^###^*P* < 0.001 for the control group vs the LX2 ESPs-CM group. **C** Expression of TGF-β1-, IL-6-, EMT- and fibrosis-related proteins. Individual bands were quantified and normalized to GAPDH. Data are presented as the mean ± SE of 3 independent experiments. Asterisks indicate a significant difference at *P < 0.05, **P < 0.01, ***P < 0.001, compared with untreated H69 cells. Con, Non-CM control; GAPDH, glyceraldehyde-3-phosphate dehydrogenase; EMT, epithelial-mesenchymal transition; ESPs, excretory-secretory products; IL, interleukin; SE, standard error; TGF-β1, transforming growth factor beta 1

The observed significant increase in TGF-β1 and IL-6 secretion levels in LX2 cells compared to H69 cells prompted an investigation of the effects of cytokines secreted by LX2 cells on H69 cells. H69 cells untreated and treated with ESPs were incubated with LX2-ESPs-CM for 24 h, following which the TGF-β1 and IL-6 secretion levels were then measured using an ELISA. As shown in Fig. 3A and B, TGF-β1 and IL-6 secretion levels were significantly increased in cells cultured in LX2-ESPs-CM compared with those in cells cultured in non-CM (TGF-β1: t-test, *t*_(4)_ = − 11.525, *P* < 0.001 and *t*_(4)_ = − 7.851, *P* = 0.00142, respectively; IL-6: t-test, *t*_(4)_ = − 11.293, *P* < 0.001 and *t*_(4)_ = − 12.335, *P* < 0.001, respectively). However, no significant differences were found in TGF-β1 and IL-6 secretion levels between H69 and ESP-treated H69 cells under LX2-ESPs-CM culture conditions.

The changes in the expression of the proteins described above in H69 cells cultured in LX2-ESPs-CM were further examined using immunoblot analyses (Fig. 3C). The expression levels of pSTAT3, pSmad2/3 and Smad4 in H69 cells cultured in LX2-ESPs-CM increased to almost the same levels as those of H69 cells treated with ESPs. Additionally, N-cad, Snail, α-SMA and fibronectin expression levels were concomitantly elevated in H69 cells grown in LX2-ESPs-CM. E-cad expression was markedly decreased in both H69 cells treated with ESPs and H69 cells grown in LX2-ESPs-CM, relative to untreated H69 cells (E-cad: t-test, *t*_(4)_ = 4.72, *P* = 0.00917 and *t*_(4)_ = 8.458, *P* = 0.00107, respectively). These results indicated that ESPs alone and secretory components of LX2 cells induced by ESPs enhanced the TGF-β1 and IL-6 secretion levels and the progression of EMT and fibrosis in H69 cells.

**Fig. 4:**
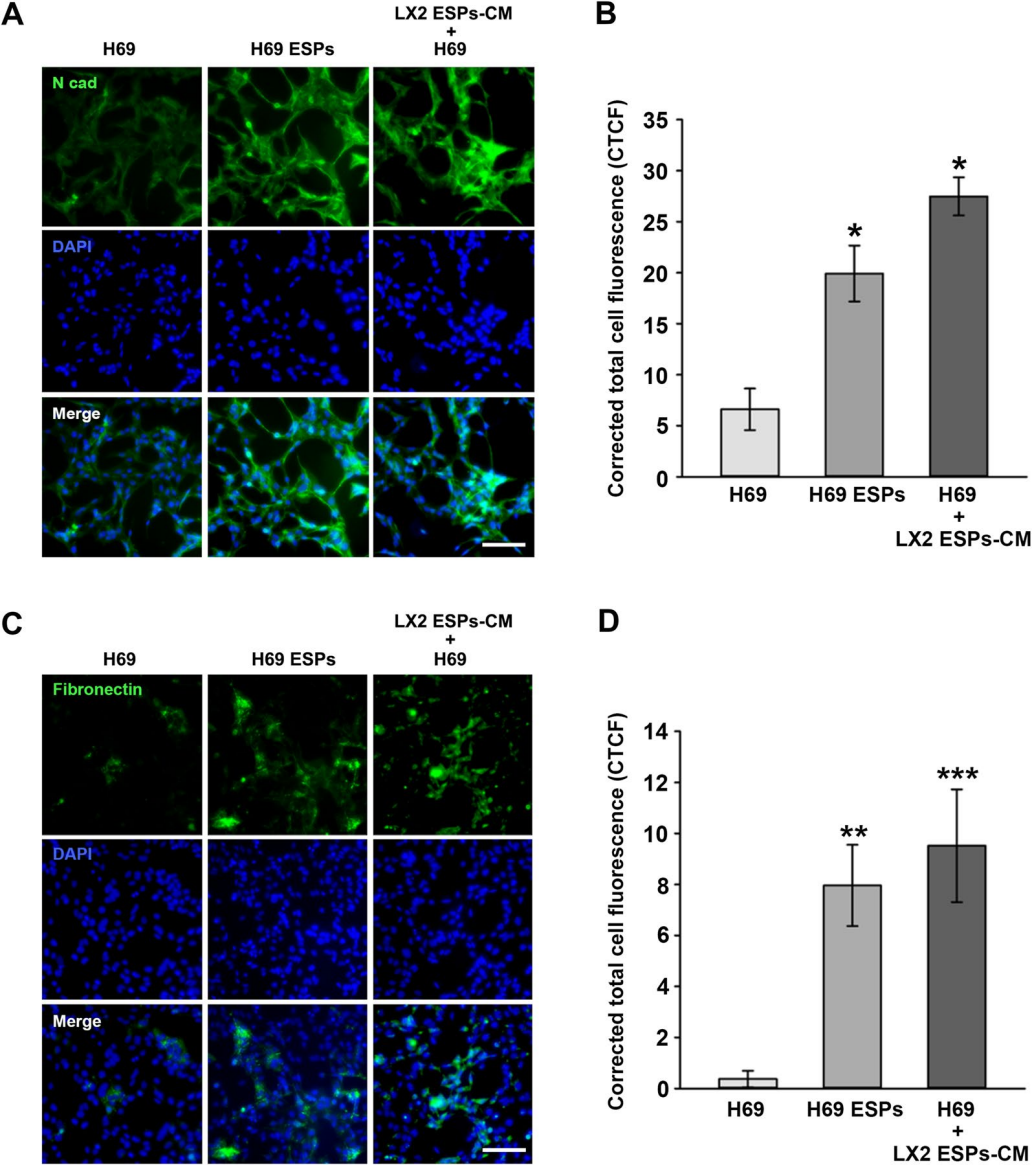
Immunostaining images of N-cad and fibronectin in H69 cells cultivated in media in which LX2 cells were grown in the presence of ESPs (LX2-ESPs-CM). **A**, **C** Representative immunofluorescence images of N-cad and fibronectin (green). DAPI was used as the nuclear counterstain (blue). Scale bar: 100 μm; original magnification: ×40. **B**, **D** Quantification of fluorescence intensity shown in** A** and** C**, respectively, using ImageJ software. Data in graphs are presented as the mean ± SE (*n* = 3). Asterisks indicate a significant difference at *P < 0.05, **P < 0.01 and ***P < 0.001, compared with untreated H69 cells. DAPI, 4′,6-Diamidino-2-phenylindole; ESPs, Excretory-secretory products; N-cad, N-cadherin; SE, standard error

To observe the immunofluorescence images in H69 cells, cells were stained with N-cad or fibronectin antibodies. CTCF values were markedly increased in cells treated with ESPs or incubated with LX2-ESPs-CM compared with those of untreated cells (N-cad: *t*-test, *t*_(4)_ = − 3.894, *P* = 0.0176 and *t*_(4)_ = − 3.969, *P* = 0.0166, respectively; fibronectin: *t*-test, *t*_(4)_ = − 4.664, *P* = 0.00956 and *t*_(4)_ = − 8.025, *P* = 0.00131, respectively) (Fig. 4). This result showed that ESPs and LX2 cells exposed to ESPs affected EMT and fibrosis progression in neighboring H69 cells.

### ESP stimuli induce H69 and LX2 cell migration through the ECM in a 3D culture model

**Fig. 5:**
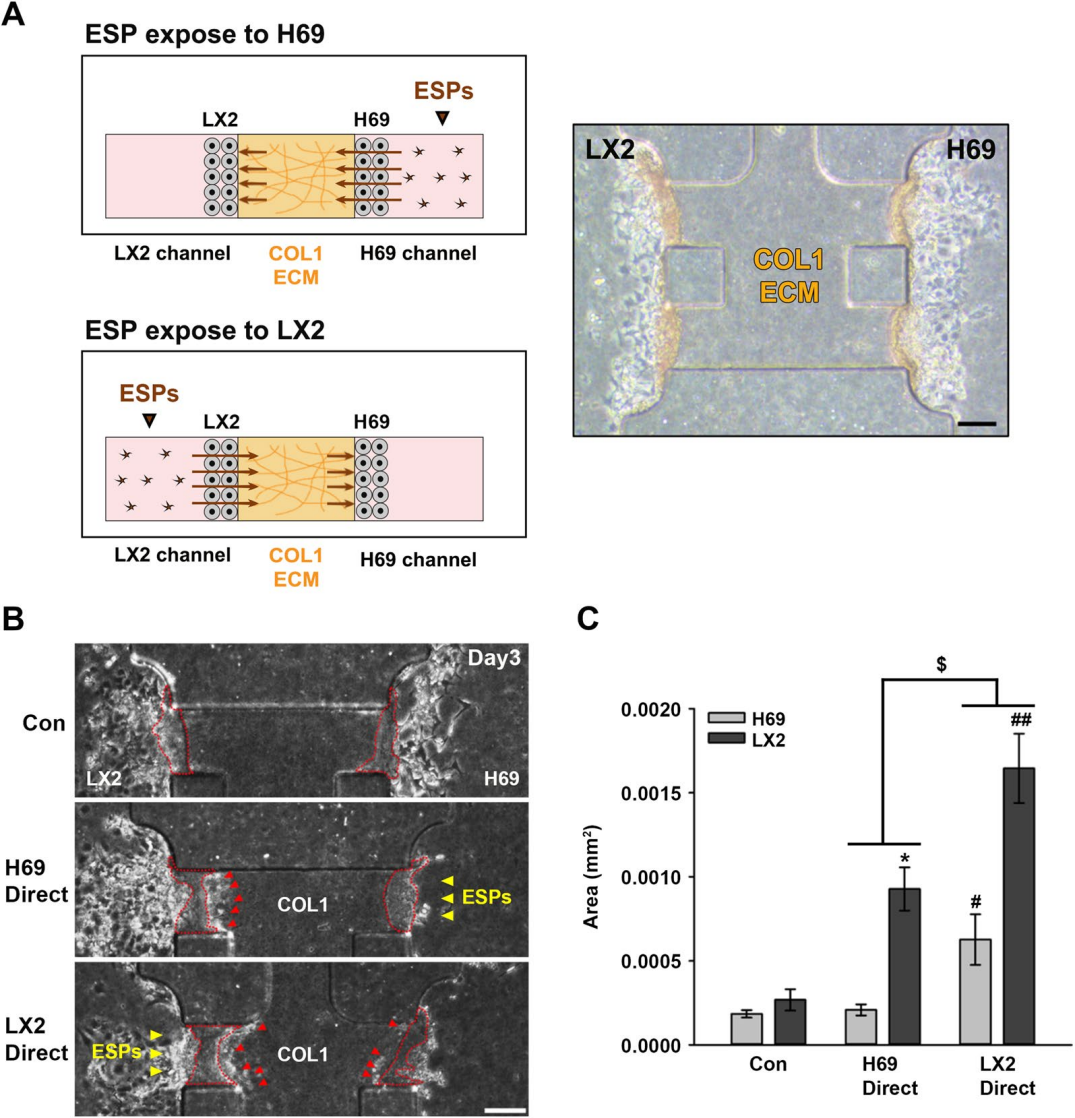
ESP-induced migration of H69 and LX2 cells cultured in a three-dimensional (3D) microfluidic device. **A** Schematic of cell culture in a 3D microfluidic device, ESP application to each channel (left), and phase-contrast image of H69 and LX2 cells cultured on both sides of the center COL1 hydrogel scaffold 24 h after seeding (right). Scale bars: 100 μm; original magnification: ×40. **B** Phase-contrast images depicting the migration of H69 and LX2 cells toward the COL1 ECM, showing penetrated area of H69 and LX2 cells in response to ESP treatment. Arrowhead indicates the direction of ESP application. CM supplemented with 4 μg/ml ESPs was applied to either the LX2 channel (left) or the H69 (right) channel, and cells were cultured for 3 days. Red dotted lines and arrowheads indicate the migrating areas of LX2 or H69 cells toward the ECM and individual ECM-penetrating cells, respectively. Scale bars: 100 μm; original magnification: ×40. **C** Quantification of LX2 and H69 cells migrating to the COL1 ECM. Data represent the mean ± SE. Single asterisk represents a significant difference at *P < 0.05, for Con vs H69 Direct; hash signs indicate a significant difference at ^#^*P* < 0.05 and ^##^*P* < 0.01, for Con vs LX2 Direct; dollar sign indicates a significant difference at ^$^*P* < 0.05, for H69 Direct vs LX2 Direct. CM, Conditioned medium; COL1, type I collagen; Con, untreated control; Con Direct, ECM, extracellular matrix; ESPs, excretory-secretory products; H69 Direct, ESPs applied to a H69 channel; LX2 Direct, ESPs applied to a LX2 channel SE, standard error

The mobility of H69 and LX2 cells mediated by the crosstalk in response to ESPs was then examined using a 3D microfluidic device that mimicked the tissue microenvironment. In a previous study, H69 cells cultured in a conditional medium (FBS-free, EGF-depleted) formed a cell layer plate structure in contact with the COL1 hydrogel scaffold in this device [18]; LX2 cells formed similar cell layer plate structures when cultured in the same conditional medium. In the present study, 1 day after cell seeding, ESPs were directly applied to H69 or LX2 cells by mixing ESPs (4 μg/ml) with conditional medium from each channel, and the cells were then incubated for 3 days (Fig. 5A). The application of ESPs to an H69 channel stimulated the penetration of LX2 cells into the COL1 ECM region; however, no penetration of H69 cells into the ECM hydrogel was detected, similar to the lack of penetration into the untreated control cells. The application of ESPs to an LX2 channel led to the penetration of both cells into the COL1 ECM, which was comparable to that of untreated or H69 cells in which ESPs were applied to the H69 channel (H69: ANOVA,* F*_(2,6)_ = 7.564, *P* = 0.0312;* F*_(2,6)_ = 7.564, *P* = 0.039; LX2: ANOVA,* F*_(2,6)_ = 22.57, *P* = 0.0013;* F*_(2,6)_ = 22.57, *P* = 0.0297) (Fig. 5B, C). The penetrated area of LX2 cells toward the COL1 ECM was approximately three- to fourfold wider than that of H69 cells, indicating that LX2 migration was associated with direct (LX2 channel) and gradient (H69 channel) ESP application, with the direct application contributing more to their migration. Additionally, components released from ESP-exposed LX2 cells influenced H69 cell migration in a 3D microfluidic device rather than the ESPs directly influencing the cells. These results suggest that ESP treatment stimulated the migratory abilities of neighboring cholangiocytes and HSCs through cell communication and ESPs.

**Fig. 6:**
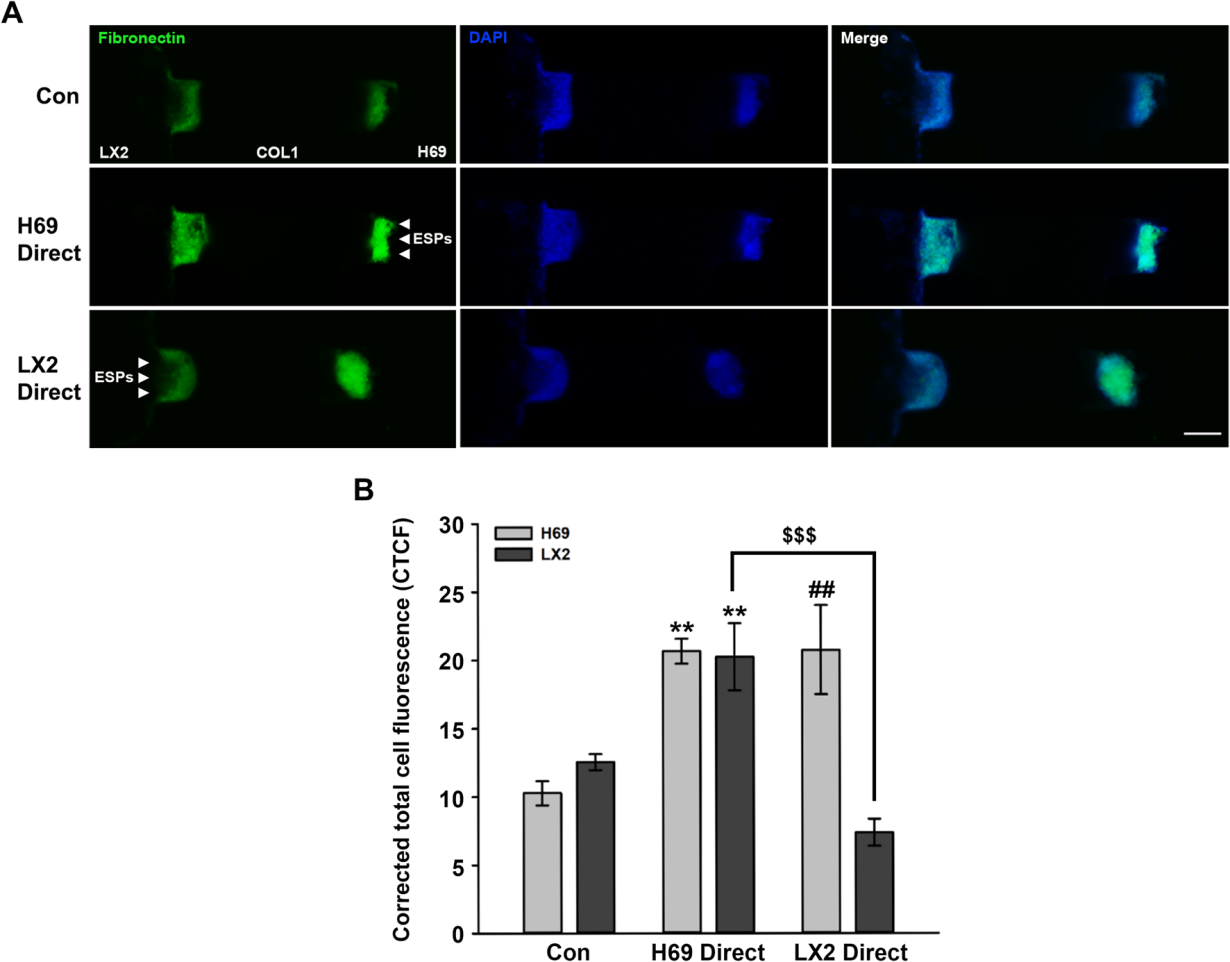
Fibronectin expression in three-dimensional-cultured cells in response to ESPs. **A** Immunofluorescence images of fibronectin expression in H69 and LX2 cells stacked on COL1 ECM. Green indicates fibronectin and blue indicates DAPI. Merged images represent the overlap of the fibronectin and DAPI. Scale bars: 100 μm; original magnification: ×40. **B **Quantification of corrected total cell fluorescence. Data represent the mean ± SE. Double asterisks indicate a significant difference at **P < 0.01, for Con vs H69 Direct; double hash signs indicate a significant difference at ^##^*P* < 0.01, for Con vs LX2 Direct; triple dollar signs indicate a significant difference at ^$$$^*P* < 0.001, for H69 Direct vs LX2 Direct. Con, Untreated control; DAPI, 4′,6-diamidino-2-phenylindole; ESPs, excretory-secretory products; H69 Direct, ESPs applied to a H69 channel; LX2 Direct, ESPs applied to a LX2 channel; SE, standard error

Immunofluorescent staining of fibronectin showed that fibronectin levels were significantly elevated in both cell types following application of ESPs to the H69 channel. The application of ESPs to an LX2 channel also increased the expression of fibronectin in H69 cells, but no obvious change in fibronectin levels was observed in LX2 cells (Fig. 6). These results suggested that ESPs and ESP-mediated cell interactions contribute to the malignant transformation of cells in the biliary microenvironment.

### Expression of N-cadherin and collagen fiber deposition in C. *sinensis*-infected mouse livers

**Fig. 7:**
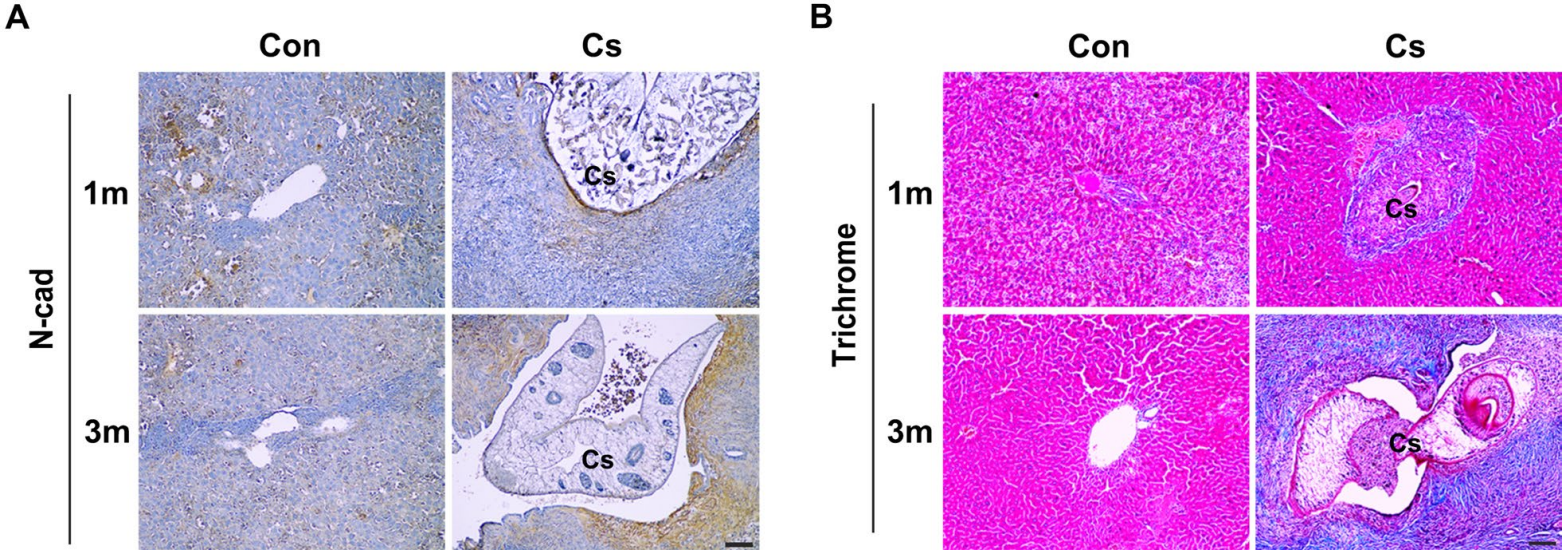
Expression of N-cad and deposition of collagen fibers in the livers of uninfected and infected mice with *Clonorchis sinensis*. Three independent liver sections from 2 infection time points were immunostained with polyclonal antibody to N-cad and stained with Masson’s trichrome for collagen fibers, respectively. Immunoreactivity of N-cad is shown in brown (**A**), and the deposition of collagen fiber is shown in blue (**B**). Scale bar: 100 μm; original magnification: ×100. Con, Uninfected control; N-cad, N-cadherin; Cs, C. *sinensis* worm

N-cad expression and hepatic fibrosis (deposition of collagen fibers), both indicators of the progression of malignant transformation, were also detected in the histopathological regions of mouse liver tissues infected with *C. sinensis* metacercariae. Intense immunoreactivity of N-cad was evident in epithelial and hyperplasia regions in the bile duct at 1 month after infection and extended to the inflammatory region at 3 months. Immunoactivity was undetectable in uninfected bile duct regions at 1 and 3 months, except for weak staining in hepatocytes (Fig. 7A). Additionally, trichrome staining exhibited no detectable collagen fibers in the livers of both uninfected controls. Collagen fibers were densely deposited in the bile duct epithelial and hyperplasia regions at 1 month. These depositions were further extended to the entire region surrounding the bile duct at 3 months (Fig. 7B). These results indicate that the upregulation of N-cad and biliary fibrosis occurred during *C. sinensis* infection, similar to in vitro ESP exposure.

## Discussion

Infection with liver fluke provokes severe pathological changes in biliary trees and their surrounding tissues, including adenomatous hyperplasia of biliary epithelia, periductal inflammation and fibrosis, and dysplasia or neoplasia of biliary and hepatic cells [6, 7]. Cellular crosstalk between bile resident and non-resident cells is crucial for the initiation and progression of pathology. In the present study, changes in pathophysiology-related protein expression and cellular interaction of two liver-constituting cells, cholangiocytes and HSCs, were investigated in response to exposure to *C. sinensis* ESPs. These cellular pathological changes triggered by ESPs were further confirmed in the livers of mice infected with *C. sinensis* metacercariae.

ESPs of liver flukes are widely used to mimic in vivo infection in a cellular microenvironment, engendering multiple pathological processes in host cells. In particular, increased secretion of inflammatory cytokines, such as TGF-β, IL-6 and tumor necrosis factor-alpha (TNF-α), have been reported in HuCCT1 cells and mouse hepatic macrophages exposed to *C. sinensis* ESPs, resulting in immunopathological responses in the host cells [22, 24]. It has also been reported that elevated plasma levels of these cytokines correlated with liver lesions in infected mice, including severe inflammation, fibrosis and cirrhosis [25]. In the present study, ESP treatment of H69 and LX2 cells resulted in increased TGF-β1 and IL-6 secretion. Concurrently, morphological changes in H69 cells to fibroblast-like cells were observed, as was increased expression of EMT- and fibrosis-related proteins in both H69 and LX2 cells (Fig. 1). Consistent with these findings, it was previously reported that *C. sinensis* infection activated the TGF-β/Smad signaling pathway, contributing to collagen deposition and subsequent liver fibrosis in infected mice [26]. Additionally, a close association between elevated plasma IL-6 concentration and increasing risk of developing advanced periductal fibrosis and CCA was reported in *O. viverrini*-infected patients [27]. Treatment with TGF-β1 or IL-6 was reported to promote the activity of EMT and invasion in biliary tract cancer cell lines [28]. In addition, TGF-β1 treatment has been found to induce mesenchymal features in CCA cell lines (CCKS-1 and TFK-1), correlated with the downregulation of E-cad mRNA expression and an increase in Snail expression [29]. Taken together, these findings suggest that aberrant regulation of TGF-β1 and IL-6 may facilitate an immunopathological environment in the biliary tracts and surrounding liver tissues during chronic clonorchiasis, ultimately promoting malignant cell transformation and even cholangiocarcinogenesis.

Cholangiocytes are the primary targets of liver fluke-associated injury, with these cells regulated and repaired in an autocrine manner. Additionally, various paracrine actions of other resident and non-resident cells are involved in the progression of inflammation, proliferation, fibrosis and even malignant transformation. In this study, exposure of H69 or LX2 cells to ESPs significantly increased the migration and invasion of co-cultured cells (LX2 or H69) at 3 days after ESP exposure, as assessed using a Transwell assay. Phenotypical changes in LX2 cells triggered by H69 cell stimulation were not observed at 1 day after ESP exposure, whereas changes in H69 cells were obvious with LX2 cell stimulation (Fig. 2). A possible explanation for this difference is that active factors released from ESP-treated LX2 cells may be more abundant than those from H69 cells, leading to prompt motile responses by H69 cells. Relatively prolonged ESP exposure may result in the accumulation of active factors secreted from H69 cells, eventually in sufficient amounts to induce LX2 cell migration and invasion. This hypothesis is supported by the results of one of our experiments: the culture of H69 cells in LX2-ESPs-CM dramatically elevated the TGF-β1 and IL-6 secretion levels of H69 cells compared with those of H69 cells directly treated with ESPs. This culture supernatant altered the expression of EMT- and fibrosis-related proteins in H69 cells so that it was almost equivalent to the regulation induced by ESPs (Figs. 3, 4). Similar to our findings, in previous studies, co-cultured HCC cells with activated HSCs-CM exhibited increased motility by activating the ERK and FAK-MMP9 signaling pathways, respectively [30, 31]. In mouse subcutaneous tumor models, COL1 secreted from activated HSCs reportedly triggers the EMT progression of HCC cells for metastasis [32]. Altogether, our findings indicate that H69 and LX2 cells differentially respond to ESPs and that bidirectional interactions between these cells synergistically enhance malignant progression, such as motility, EMT and fibrosis, in the biliary microenvironment.

A microfluidic 3D cell culture assay system was developed to mimic different types of in vivo cellular microenvironments. This system comprises cells cultured in microfluidic channels incorporating an ECM hydrogel scaffold and biochemical and fluidic components, which precisely control the conditions for cell–cell and cell-ECM interactions [20]. Recently, a 3D microfluidic co-culture system has been applied to design an in vitro lymphangiogenesis model in a tumor microenvironment, mediated by the communication between cancer and lymphatic endothelial cells [33]. Similar to our previously established in vitro clonorchiasis-associated tumor model [18], a modified 3D microfluidic co-culture system was used in the present study to examine the effect of ESP-exposed cells on neighboring cells in a biliary microenvironment. This system comprised two cells (H69 and LX2 cells) separated by a COL1 ECM and the channels of ESP application. ESP application to the H69 channel caused increased penetration of LX2 cells into ECM hydrogels (Fig. 5), which is consistent with the result of the above-mentioned Transwell assay and previous findings that various factors released from reactive cholangiocytes contributed to HSC activation and migration [34, 35]. In comparison, ESP application from the LX2 channel induced motile activities of H69 and LX2 cells toward the ECM (Fig. 5). It is possible that various factors secreted from ESP-exposed LX2 cells not only affect their motilities in an autocrine manner but also affect neighboring H69 cells in a paracrine manner. This possibility is supported by our results that the basal and ESP-induced levels of TGF-β1 and IL-6 in LX2 cells were much higher than those in H69 cells (Fig. 1). Additionally, it has been reported that stellate cells play major roles in ECM degradation by upregulating the activities of matrix metalloproteinases (MMPs) [36]. The MMP-2 messenger RNA (mRNA) expression was increased in TGF-β1-treated HSCs [37]. Therefore, it is plausible to speculate that TGF-β1 and IL-6 released from HSCs may sufficiently penetrate the ECM degraded by MMPs, contributing to the stimulation of neighboring H69 cell motility. With regard to H69 cells, the expression of MMP-9 mRNA and protein was reported to be barely detectable even through ESP treatment, compared with those of HuCCT1 cells [23], explaining the poor invasive capability of H69 cells toward ECM. Concomitantly, we observed increased fibronectin expression levels in migrating cells in the 3D culture (Fig. 6), implying that ESPs provoke chemoattraction of proximal cells in biliary trees and that dysregulated interactions between these cells promote pathophysiological progress such as fibrosis.

Dysregulated expression of proteins involved in EMT and fibrosis has been observed in various types of hepatobiliary cancer tissues. For example, N-cad expression was dramatically elevated in intrahepatic CCA tissues from patients [38]. The change in decreasing E-cad and increasing N-cad expression (cadherin switch) is a strong prognostic factor for extrahepatic CCA [39]. A strong expression of COL1 was observed in the livers of patients with opisthorchiasis-associated CCA [40]. Differential accumulation of collagen subtypes in a *C. sinensis*-associated hamster CCA model suggested that these may be involved in CCA initiation and progression [9]. An increase in N-cad expression and collagen fiber deposits was found in and around the bile duct of *C. sinensis*-infected mouse liver tissue, with the intensity of both increasing in a time-dependent manner and extending to the surrounding bile duct region (Fig. 7). Overlapping N-cad overexpression with collagen fiber deposition in the periductal fibrotic region of infected livers implies that fibrotic progression may be associated with the local induction of an EMT process. This was supported by the finding that stimulation of primary intrahepatic biliary epithelial cells with TGF-β1 induced EMT marker expression. Also, biliary epithelial cells underwent EMT, contributing to portal tract fibrosis in the liver sections of numerous chronic cholangiopathy patients [41]. These findings raise the possibility that EMT occurs during periductal fibrosis in *C. sinensis*-infected livers, which plays a detrimental role in the increased severity of hepatobiliary abnormalities. Therefore, it is of interest to find the profibrotic mediators and to examine the pathophysiological processes of functional cholangiocytes or/and HSC into myofibroblasts during *C. sinensis* infection.

## Conclusions

In conclusion, *C. sinensis* ESP treatment modulated the expression of EMT- and fibrosis-related proteins and the secretion of TGF-β1 and IL-6 in H69 and LX2 cells. Mutual interaction between these cells affected the expression of cytokines and proteins involved in EMT and fibrosis, driving cell migration and invasion in an ESP-treated microenvironment. Increased N-cad expression and collagen fiber deposition in *C. sinensis-*infected mouse livers indicate that EMT is closely associated with periductal fibrotic progress. These processes further promote malignant transformation leading to the development of advanced hepatobiliary diseases, including cholangiocarcinogenesis. These findings broaden our understanding of the cellular crosstalk between cholangiocytes and HSCs for EMT and biliary fibrosis development and progression during *C. sinensis* infection. The results of this study will allow for the identification of potential therapeutic targets to reduce clonorchiasis-associated biliary fibrosis.

## Data Availability

All data generated or analysed during this study are included in this published article.

## Abbreviations

α-SMA: Alpha-smooth muscle actin
CCA: Cholangiocarcinoma
CM: Conditional medium
ESPs: Excretory-secretory products
EMT: Epithelial-mesenchymal transition
E-cad: E-cadherin
HCC: Hepatocellular carcinoma
HSCs: Hepatic stellate cells
IL: Interleukin
N-cad: N-cadherin
TGF-β1: Transforming growth factor beta 1
